# Philadelphia Academy of Surgery. Meeting April 3, 1893. The President, Dr. William Hunt, in the Chair

**Published:** 1893-08

**Authors:** John H. Brinton

**Affiliations:** Philadelphia


### PHILADELPHIA ACADEMY OF SURGERY.

MEETING APRIL 3, 1893.

The PRESIDENT, DR. WILLIAM HUNT, in the chair.

### TWO CASES OF AMPUTATION AT SHOULDER-JOINT IN WHICH WYETH'S PINS, TO CONTROL HEMORRHAGE, WERE USED.

BY JOHN H. BRINTON, M. D., PHILADELPHIA.

CASE I. *Osteitis deformans of the leg, followed at the expiration of twenty-three years by sarcoma of the humerus; amputation at shoulder-joint by the oval method; use of Wyeth's pins to control hemorrhage; death on the tenth day.*—E. B., aged forty-eight years; born in Massachusetts; publisher. About twenty-three years ago he noticed tenderness over the right tibia, increased by pressure, by severe or prolonged exercise, and by barometric changes. Various anti-rheumatic measures were employed, but without avail. The limb did not become much worse; he was able to be about and follow the business of his life for years. During this time he was not lame, but experienced a sense of weakness in the limb. To use his own expression, "he favored that leg." In the course of years the bones of the leg had gradually increased in thickness, and had become curved. About ten years ago he consulted the late Prof. Agnew, who told him that he could do nothing to relieve his slight disability of the limb, and that the affection was incurable. About three years since he consulted me, but I could add nothing to what had been already said and could suggest no treatment.

In November, 1891, the patient consulted me for a fracture of the body of the left scapula. This resulted from a fall backward, as he was descending from the step of a railroad car, the scapula striking the edge of a projecting board or plank. This fracture healed rapidly and well.

In June, 1892, in jumping from a street car while in motion, and while his hand grasped the railing, he experienced great pain just below the right shoulder, and felt that the arm was broken. He came directly to my office. On examination, I detected crepitus, diagnosticated fracture of the anatomical neck of the humerus, and treated him for that injury. Union took place quickly, and full use of the limb was obtained. The only noticeable feature in this injury was the occurrence of slight pain referred to the outside of the humerus, about the lower portion of the upper third. There was at that time no enlargement of the bone at this locality.

On September 19, 1892, the patient again consulted me, stating that a "lump" had appeared on the outside of the humerus at its upper part. I examined the arm and found distinct cylindrical enlargement of the humerus, obviously a sarcoma of the bone, and I stated this to the patient, advising him to consent to the removal of the limb at the shoulder-joint, if the diagnosis should be confirmed by a preliminary incision. At the patient's request, Drs. Packard and John Ashhurst saw the case in consultation, and they agreed with me in the propriety of immediate operation. From a careful examination of the patient's entire clinical history, there was no doubt in our mind that the case was one of osteitis deformans, first described by Paget, and which had been followed, as is so often the case, by the development of a malignant growth.

The operation was fixed for October 5, 1892, but at the preliminary shaving of the axilla, and preparation of the limb, or by the patient's lifting of the limb, fracture, which may fairly be regarded as spontaneous and non-traumatic, occurred, as was made evident at the time of operation, done in the presence and with the assistance of Drs. Keen, Ashhurst, Packard, and others.

To prevent hemorrhage, the long steel pins of Prof. Wyeth were inserted by Dr. Keen, the anterior one transfixing the anterior axillary fold in front of the vessels, penetrating the tendon of the pectoralis major muscle, and emerging near the end of the acromion.

The posterior needle pierced the deltoid and emerged just below the acromion. By carrying the needle, especially the anterior one, well upward, the constricting rubber band was placed so high as not to prevent the rotation of the humeral head, or to interfere materially with its disarticulation.

This patient suffered very slight loss of blood at the time of the operation, and received but little shock. He reacted promptly and perfectly, and for several days did well, the wound uniting throughout. On the night between the fifth and sixth day the temperature rose to 104.5 degrees, and a copious eruption, similar to that of measles, appeared on the abdomen and chest, and eventually invaded the extremities, and indeed the whole body. There was marked coryza, and the tongue became brown and dry. This condition resisted all treatment and the free use of antipyretics. As the eruption spread, the temperature still rose, reaching 107.5 degrees and 108 degrees, and the patient died on the afternoon of the 15th of October, the tenth day after the operation. The intellect remained clear until within an hour or so of the end.

I cannot but regard the death as due to some form of septic infection not easy to determine.

It is unnecessary to add that the antisepsis was observed in the treatment before, during, and after the operation.

The specimens, showing the sarcoma of the shaft of the humerus, and the peculiar indented fracture of its head and anatomical neck, are before this Academy. I particularly desire the observation of the Fellows to the fracture, which appears to me to have resulted from violent impact of an infiltrated diseased caput humeri against the edge of the glenoid cavity.

CASE II. *Amputation at shoulder-joint for enchondroma of humerus.*—The other case of shoulder amputation, in which I used Wyeth's pins, was that of a boy (I. B.), from Vermont, ten years of age. Nearly a year previously a tumor, apparently an enchondroma, began to develop on the inner side of the humerus, close to the head of the bone. It eventually grew until it attained a diameter of two and a half inches. It was painless, but interferred with the joint-motion by its bulk. The boy was brought to the clinic of the Jefferson Hospital, and after consultation with my colleagues, I determined to remove the arm at the shoulder.

This was accordingly done on the 28th of November, Wyeth's pins being first introduced by my colleague, Professor Keen. The anterior pin was made to emerge three-quarters of an inch above the tip of the acromion. As a result the circular turns of the tubing rested on a somewhat higher level than in the preceding case. Perfect freedom of the joint was preserved and its disarticulation was not impeded. Previous section of the bone with the saw, as directed by Professor Wyeth, was not necessary. A roller bandage was applied as a compress under the tubing and directly over the artery. Hemorrhage was thus perfectly prevented, and the removal of the limb, as in the former case, was practically a bloodless procedure. This boy recovered without accident.

I may state that in both these instances an Esmarch elastic bandage was applied previous to the insertion of the pins.

DISCUSSION.

DR. WILLIAM W. KEEN: I would call attention to the control of hemorrhage by Wyeth's method. This afforded perfect hæmostasis. I never saw anything better, and as compared with the method which I devised myself a few years ago, by a compress over the subclavian artery, I think that it is vastly superior. In the first of Dr. Brinton's cases the pins were brought out at the end of the acromion process, and when the head of the bone was removed the skin slipped down and the constriction of the tube partially obliterated the cavity where the head of the bone had been. In the second case, the pins emerged three-fourths of an inch from the tip of the acromion, and there was no trouble from the slipping of the tube downward.

DR. JOHN B. DEAVER: How much blood was lost in these cases?

DR. BRINTON: In the first case there was a little blood lost on account of the slipping of the rubber tube—perhaps two ounces. In the second case there was practically no blood lost.

DR. DEAVER: I have had no experience with the Wyeth pins in amputation at the shoulder-joint. I have relied upon good assistance and have never seen much bleeding. There is no doubt that the method described is an excellent one, and the only question in my mind was whether the presence of the tube did not interfere with the manipulation in disarticulating. In the few chronic cases on which I have operated all have recovered.

DR. JAMES M. BARTON: Unless there is some tumor encroaching upon the joint, interferring with the manipulation, I am in favor of an assistant grasping the artery as the flap is divided. Some years ago I saw a case of large sarcoma of the head of the humerus perish from hemorrhage on the table. The pins under such circumstances would have saved life.

DR. W. W. KEEN: I think that there is no possible doubt that the tube does not interfere with the manipulations, but that it assists us in making them. You have absolute confidence in your hæmostasis which you cannot have in any assistant whose arm or thumb is apt to get tired.

DR. H. R. WHARTON: I have not had any experience with Wyeth's pins in shoulder-joint amputation, but I can see how the method should be very useful, although I have not seen much bleeding where there have been good assistants. In my experience the most blood has been lost in the preliminary incisions. The only point would be in regard to the interference with the disarticulation if the tube slipped. I have seen Dr. Agnew use a pin under the vessels with a ligature above, which controlled the hemorrhage very satisfactorily.

THE PRESIDENT: Some years ago I used the Esmarch tube in the of a figure of eight in amputation at the shoulder-joint with perfect success. An assistant held it up when there was any tendency to slip.

